# 

**Published:** 1916-11-04

**Authors:** 


### HOSPITAL AND INSTITUTIONAL RESULTS IN 1915.

**Royal Sussex County Hospital, Brighton.**—The total income, including £5,932 legacies, was £19,191, and the total expenditure was £18,846. The last figure showed a decrease on the previous year, when, however, there was heavy extraordinary expenditure. Although the war work was unusually heavy, all the nurses presenting themselves passed the medical and surgical examinations. Six hundred and forty-two military cases, including eleven cases of military nurses who had broken down in their work, were received; at the end of the year there were seventy-one soldiers in the wards.

**Great Northern Central Hospital.**—The annual report, the distribution of which, it will be remembered, was for reasons of economy limited to those who expressed a wish to receive it, has now reached us. It appears to have been prepared in the customary way, includes about 200 pages, eight of which are illustrations on art paper, and a map of the districts served by the hospital. The accounts show a surplus of income for the year of £443, but this, we gather from the jubilee appeal, has now been turned into an adverse of about £8,000. Up to the end of the year 783 military cases had passed through the wards.

**Bolton Infirmary and Dispensary.**—In our issue of June 17 the suggestion was made that the accounts might with advantage be presented as from January 1 to December 31; this step, we rejoice to learn, will be taken after the current year, and this important institution will thus fall into line with the vast majority of the hospitals of the country. For the twelve months ending February 29, 1916, the expenditure exceeded the income by £840. The ordinary expenditure was £1,352 more than in the previous financial year.

### TWO WAR HOSPITAL GAZETTES.

The gazette of the 1st Eastern General Hospital, Cambridge, which is published twice a month, contained in its first issue for October a reminder of the approach of winter in its statements as to the closing-in of the wards. It contains, not for the first time, if we remember, several parodies, notably one on Browning, and a sketch or two. The verse keeps closer to hospital subjects than the prose, but neither is very ambitious or remarkable.

By merit, rather than by choice, pride of place in any notice of London war hospital magazines must be given to the gazette of the 3rd London General Hospital, Wandsworth, which began a second volume with its October number. We have said, perhaps too often, that the best material for hospital gazettes is provided by incidents of daily occurrence, observations of character, an account of his or her work by every member of the staff, no matter of what position, who has the faculty of self-expression. The October number of the *3rd London Gazette* is an instance of this, and no better could be offered. But it is not every hospital which began with a staff of artists.

Rates of Subscription: Twelve months, 6/6; Six months, 4/-; Three months, 2/-; post free to any address in the United Kingdom. Abroad, Twelve months, 8/8; Six months, 5/-; Three months, 2/6, post free. The Hospital "Index" to Volume LX., April to September 1916, is now ready, price 2½d. per copy, post free. Address The Manager, "The Hospital," The Hospital Building, 28/29 Southampton Street, Strand, London, W.C.

### Publications.

By SIR WILLIAM BENNETT, K.C.V.O., F.R.C.S.

***Crown 8vo. 5s. net.***

**INJURIES AND DISEASES OF THE KNEE JOINT**

With 34 Illustrations.

NISBET & CO.

***Also, 8vo. 6s.***

FOURTH EDITION.

**Massage and Movements in Recent Fractures; Sprains and their Consequences; Rigidity of the Spine and the Management of Stiff Joints.**

With 28 Illustrations.

LONGMANS, GREEN & CO.

**NEW EDITION in Two Vols. 45s. net.**

**DEFORMITIES,**

INCLUDING

**DISEASES of the BONES and JOINTS.**

A Text-Book of Orthopædic Surgery.

Illustrated by 70 Plates and over 1,000 Figures and by Notes of Cases.

By **A. H. TUBBY, M.S.Lond., F.R.C.S.Eng.**

"The standard text-book in English on the subject."—*Lancet.*
First Edition. "Standard work on the subject in the English language." *British Medical Journal.*

**MACMILLAN & CO., LTD., St. Martin Street, London, W.C.**

2nd Edition. viii+308 pages. Price **6s.** net.

**THE TREATMENT OF DISEASES OF THE SKIN.**

By W. KNOWSLEY SIBLEY, M.A., M.D., B.C. (Camb.), M.R.C.P., Physician to St. John's Hospital for Diseases of the Skin, London. Illustrated with 30 Original Photographs and 150 Special Prescriptions.

LONDON: EDWARD ARNOLD, 41 & 43 MADDOX STREET, W.

8vo., 12s. 6d. net. Stereoscope, 2s. 6d. net.

**RHINOLOGY:**

**A Text-Book of the Diseases of the Nose and Accessory Sinuses.**

47 *Plates and* 146 *Illustrations.*

By P. WATSON-WILLIAMS, M.D. Lond.,

Lecturer on Diseases of Ear, Nose and Throat, University of Bristol, and in Charge of Ear, Nose, and Throat Department, Bristol Royal Infirmary.

LONGMANS, GREEN & CO., 39 Paternoster Row, London, E.C.

